# GCN2 Has Inhibitory Effect on Human Immunodeficiency Virus-1 Protein Synthesis and Is Cleaved upon Viral Infection

**DOI:** 10.1371/journal.pone.0047272

**Published:** 2012-10-23

**Authors:** Javier del Pino, José Luis Jiménez, Iván Ventoso, Alfredo Castelló, Ma. Ángeles Muñoz-Fernández, César de Haro, Juan José Berlanga

**Affiliations:** 1 Centro de Biología Molecular Severo Ochoa (CSIC-UAM), Universidad Autónoma de Madrid, Madrid, Spain; 2 Plataforma de Laboratorio, Hospital General Universitario Gregorio Marañón, Madrid, Spain; 3 Laboratory of Molecular Immunobiology, Hospital General Universitario Gregorio Marañón, Madrid, Spain; Institut National de la Santé et de la Recherche Médicale, France

## Abstract

The reversible phosphorylation of the alpha-subunit of eukaryotic translation initiation factor 2 (eIF2alpha) is a well-characterized mechanism of translational control in response to a wide variety of cellular stresses, including viral infection. Beside PKR, the eIF2alpha kinase GCN2 participates in the cellular response against viral infection by RNA viruses with central nervous system tropism. PKR has also been involved in the antiviral response against HIV-1, although this antiviral effect is very limited due to the distinct mechanisms evolved by the virus to counteract PKR action. Here we report that infection of human cells with HIV-1 conveys the proteolytic cleavage of GCN2 and that purified HIV-1 and HIV-2 proteases produce direct proteolysis of GCN2 in vitro, abrogating the activation of GCN2 by HIV-1 RNA. Transfection of distinct cell lines with a plasmid encoding an HIV-1 cDNA clone competent for a single round of replication resulted in the activation of GCN2 and the subsequent eIF2alpha phosphorylation. Moreover, transfection of GCN2 knockout cells or cells with low levels of phosphorylated eIF2alpha with the same HIV-1 cDNA clone resulted in a marked increase of HIV-1 protein synthesis. Also, the over-expression of GCN2 in cells led to a diminished viral protein synthesis. These findings suggest that viral RNA produced during HIV-1 infection activates GCN2 leading to inhibition of viral RNA translation, and that HIV-1 protease cleaves GCN2 to overcome its antiviral effect.

## Introduction

The control of protein synthesis is central to the global process of regulation of gene expression, first leading to translational reprogramming and, as a consequence, affecting the transcriptional profile of cells. Protein synthesis is basically regulated at the initiation step, where phosphorylation of the alpha subunit of initiation factor eIF2 (eIF2alpha) at residue Ser-51 by specific protein kinases represents one of the best-characterized mechanisms regulating mRNA translation in eukaryotic cells in response to various stress conditions, such as lack of nutrients, endoplasmic reticulum stress, iron deficiency, heat shock and viral infection [1], [2]. In mammalian cells, four different eIF2alpha kinases regulated by specific signals have been identified: HRI (iron deficiency) [3], [4]; PKR (double-stranded RNA produced in cells infected by viruses) [5]; PERK (stress situations in the endoplasmic reticulum) [1]; and GCN2 (amino acid or serum deprivation and ultraviolet light irradiation) [6], [7], [8]. Some members of this family of eIF2alpha kinases are also present in other eukaryotic organisms: PERK and GCN2 in *Drosophila melanogaster* [5], [9], [10]; GCN2 in the yeast *Saccharomyces cerevisiae* [11] and two eIF2alpha kinases related to mammalian HRI (Hri1 and Hri2), in addition to Gcn2, in the fission yeast *Schizosaccharomyces pombe* [12].

eIF2 forms a ternary complex with GTP and the initiator methionyl-tRNA, which delivers the latter to the small ribosomal subunit with the subsequent hydrolisis of GTP. The conversion of inactive eIF2-GDP into active eIF2-GTP is catalyzed by the guanine nucleotide exchange factor eIF2B. Phosphorylation of eIF2alpha at Ser-51 increases its affinity for eIF2B, which then becomes sequestered in a stable and inactive complex, leading to the inhibition of protein synthesis due to the lack of ternary complexes.

GCN2 was first described in the budding yeast *Saccharomyces cerevisiae* where the kinase is activated in response to amino acid starvation through the binding of uncharged tRNA to a region homologous to the histidyl-tRNA synthetases (HisRS) [13]. In mammals, this region is also responsible for the in vitro activation of GCN2 by tRNA and viral RNA [14]. Thus GCN2 has been involved in the antiviral response against RNA viruses, such as Semliki Forest virus, vesicular stomatitis virus and Sindbis virus (SV), whose genomic RNA is able to bind and activate the kinase, which through the phosphorylation of eIF2alpha inhibits the translation of the genomic mRNA of SV and blocks its replication cycle in cells [14]. It is well known the central role of PKR in the cellular antiviral response as well as the different strategies developed by distinct viruses in order to counteract the negative effects of PKR on virus replication. These evasion strategies include proteins that inhibit PKR, sequester dsRNA, or are pseudosubstrates, and RNA molecules acting as pseudoactivators that compete with activator dsRNA for the binding to PKR [15]. Thus, during HIV-1 infection, PKR is first transiently activated and then inhibited due to viral and virus-activated cellular mechanisms of control [16], [17].

Many viruses have evolved mechanisms that modify the activity of cellular translation factors in order to favor viral mRNA translation to the detriment of cellular mRNA translation. The best-characterized example of that is the proteolityc cleavage of eIF4G by proteases of different viruses, leading to the inhibition of capped cellular mRNA translation and to the enhancement of translation of uncapped viral RNAs [18]. Thus, eIF4G is cleaved by 2A protease of rhinovirus or poliovirus, L protease of aphthovirus, and proteases of several retroviruses, including HIV-1 and HIV-2 [19], [20], [21]. Another protein involved in translation, PABP, is also a target for viral proteases, including 2A and 3C of enterovirus [22], [23], and the proteases of HIV-1 and HIV-2 [24].

HIV-1 protein translation occurs late in the viral life cycle in the cytoplasm of cells and is carried out by the host protein synthesis machinery. HIV-1 protease (HIV-1^Pro^) is a small enzyme that mediates the cleavage of Gag, Gag-Pol, and Nef precursor polyproteins during virion assembly and maturation [16].

In this paper we show that HIV-1 RNA activates GCN2 in vivo and in vitro and that the cleavage of the kinase by HIV-1^Pro^ leads to its inactivation. Synthesis of HIV-1 proteins in cells transfected with a plasmid encoding the viral RNA increased in cells devoid of the GCN2 gene. All these results suggest that GCN2 is involved in the cellular antiviral response against HIV-1 infection.

## Results

### HIV-1 RNA promotes GCN2 activation in vitro through its HisRS-related domain

**Figure 1:**
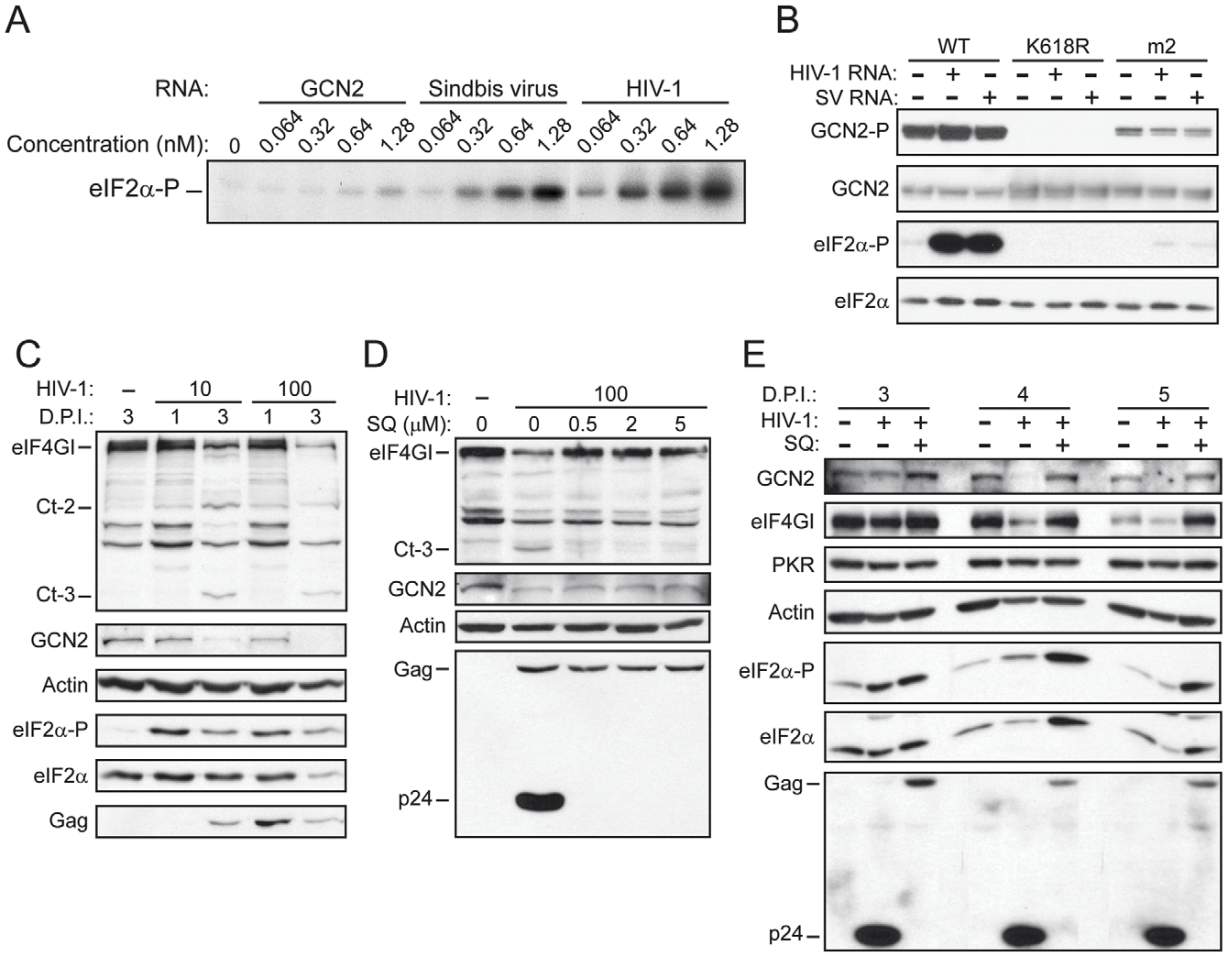
HIV-1 RNA activates GCN2 and the kinase is proteolytically cleaved during HIV-1 infection. (A) Purified mouse GCN2 was subjected to in vitro eIF2alpha kinase assay in the presence of increasing concentrations of in vitro transcribed RNAs corresponding to the mouse GCN2 open reading frame (as a negative control), or to the genomic RNAs of Sindbis virus or HIV-1, respectively, as indicated. In this experiment ^32^P-γ-ATP was included in the kinase reaction mix and phosphorylation of eIF2alpha was visualized after analysis by SDS-PAGE and autoradiography. (B) In vitro eIF2alpha kinase assay of purified wild type GCN2 (WT), GCN2-K618R (K618R) or GCN2-m2 (m2) in the presence of Sindbis virus (SV) or HIV-1 RNA, as indicated. Results were obtained by western blot analysis of the samples using different antisera to detect eIF2alpha phosphorylated on serine 51, total eIF2alpha and phosphorylated and total GCN2. (C) MT-2 cells were mock infected (−) or infected with 10 or 100 ng of p24^gag^ per 10^6^ cells of HIV-1_NL4–3_ and harvested at the indicated days post-infection (D.P.I.). Aliquots of cell lysates containing equal amount of proteins were analyzed by western blot (8% SDS-PAGE) using specific antibodies as indicated. (D) MT-2 cells were mock-infected (−) or infected with 100 ng of HIV-1 NL4-3 per 10^6^ cells, in the absence or the presence of the indicated concentrations of saquinavir (SQ) and harvested at day 3 post-infection. Aliquots of cell lysates containing equal amounts of proteins were analyzed by immunoblot (10% SDS-PAGE) using specific antibodies as indicated. (E) PHA-stimulated PBMCs were mock-infected (−) or infected with 100 ng of HIV-1 NL4-3 per 10^6^ cells, in the absence or the presence of 0.5 µM SQ and harvested at the indicated D.P.I. Aliquots of cell lysates containing equal amounts of proteins were analyzed by immunoblot using specific antibodies as indicated. Results are representative of at least three independent experiments.

Given that the genomic RNA of Sindbis virus activated GCN2 in vitro and that GCN2 have an antiviral effect against this virus [14], we tested whether the genomic RNA of other viruses could activate GCN2 as a sign of a potential antiviral effect against them. We observed that in vitro transcribed HIV-1 genomic RNA, among others, was able to significantly increase the eIF2alpha kinase activity of affinity purified GCN2 in a dose-dependent manner (Figure 1A). This activation was dependent on its HisRS-related domain, as the GCN2-m2 mutant of this domain showed very low autophosphorylation and eIF2alpha kinase activity compared to that of the wild type protein (Figure 1B). As expected, no autophosphorylation or eIF2alpha kinase activity was found when using the GCN2 form bearing a mutation in the lysine residue of the kinase domain responsible for the ATP binding (K618R). GCN2 activation by HIV-1 RNA was comparable to that obtained by using Sindbis virus RNA, which served as a positive control in these assays.

### GCN2 cleavage in cells after HIV-1 infection

In order to investigate the putative role of GCN2 in the cellular antiviral response against HIV-1, we tested GCN2 activation in MT-2 (human T-lymphocyte cell line) cells infected with HIV-1. To our surprise, the analysis of the cell lysates revealed the disappearance of GCN2 upon HIV-1 infection coinciding with the cleavage and disappearance of full length eIF4GI. Both protein bands were apparently intact at day 1, but disappeared at day 3 after infection, and their cleavage was prevented in presence of the specific inhibitor of the HIV-1 protease (HIV-1^Pro^) saquinavir (Figure 1 C and D). In the case of GCN2 we were unable to detect any proteolytic fragment, but the cleavage of eIF4GI rendered the expected carboxi-terminal Ct-2 (about 102 kDa) and Ct-3 (about 57 kDa) fragments [20]. Moreover, infection of Peripheral Blood Mononuclear Cells (PBMC) with HIV-1 resulted in a significant proteolytic degradation of GCN2 (45% reduction) and eIF4GI at 4 and 5 dpi, which was prevented in the presence of saquinavir (Figure 1E), strongly suggesting that the low levels of these proteins present in the cells were a consequence of HIV-1^Pro^ cleavage. The level of other proteins such as PKR, actin or eIF2alpha remained unaltered to the same extent throughout the experiment. Note that in Figure 1 D and E, the HIV-1 p24 protein only appeared in the absence of saquinavir as a consequence of HIV-1^Pro^ processing of the Gag (55 kDa) precursor. We also observed increased phosphorylation of eIF2alpha upon HIV-1 infection (Figure 1 C and E), suggesting a possible activation of GCN2.

### HIV-1 protease directly cleaves GCN2

**Figure 2:**
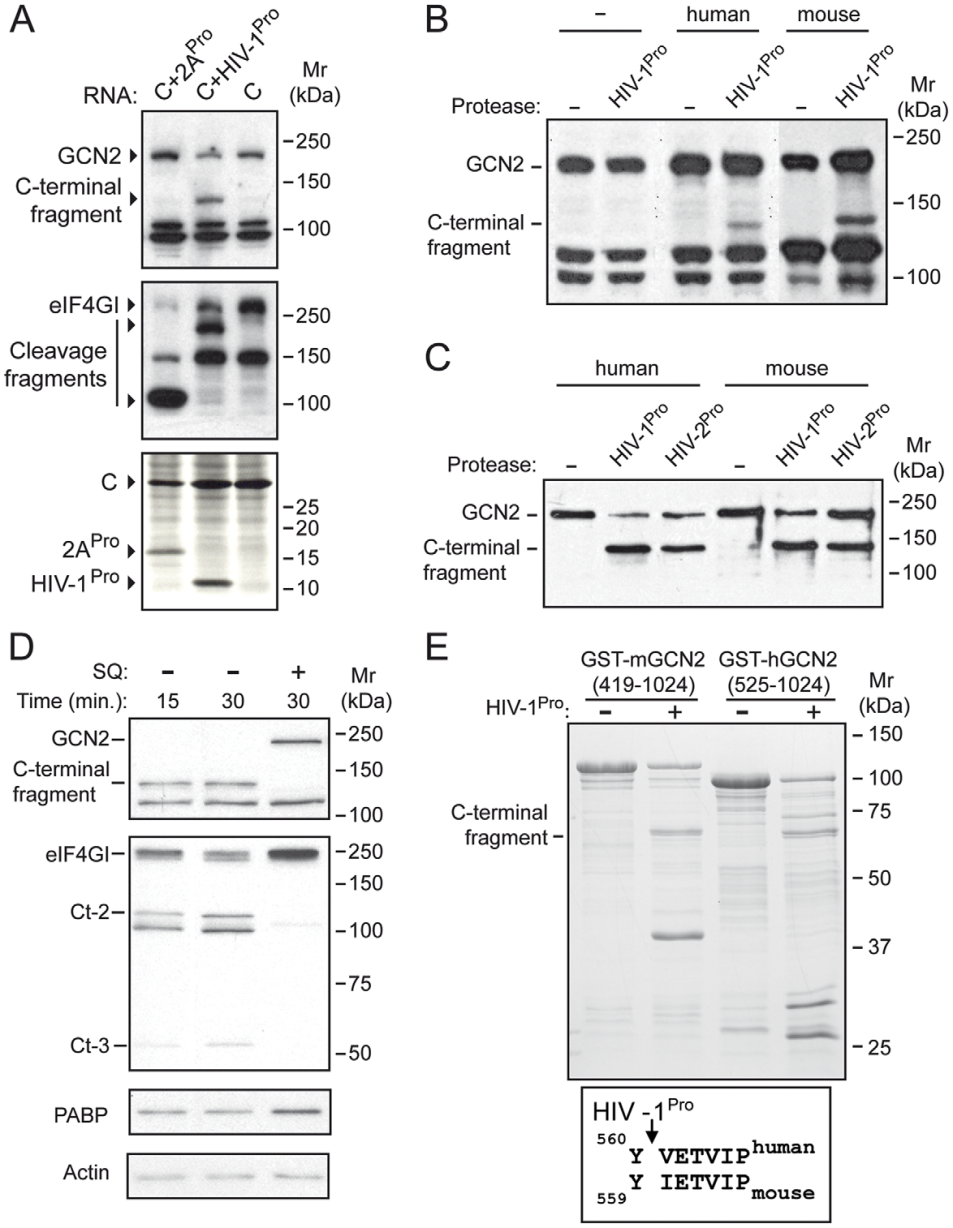
HIV-1 protease directly cleaves GCN2. (A) BHK-21 cells were transfected by electroporation with in vitro transcribed capped Sindbis virus RNAs from plasmids pT7SV-HIV-1PR (C+HIV-1^Pro^), pT7SV-2Apro (C+2A^Pro^), pT7SVwt (C) for the expression of HIV-1 and PV-2A proteases. After 4 h cells were metabolically labeled with [^35^S]-Met-Cys. Equivalent amounts of total protein were subjected to 12% SDS-PAGE, transferred to PDVF membranes and subjected to autoradiography (lower panel) to visualize protease expression. The membranes were then probed with specific antisera for detection of endogenous GCN2 and eIF4GI as indicated. (B) COS-7 cells were subjected to coupled infection/DNA transfection with recombinant vaccinia virus (vvT7), pTM1 (−) or pTM1-derived plasmid encoding HIV-1^Pro^ and pcDNA3.1/Myc-His plasmid, empty (−) or encoding human or mouse GCN2. At 18 h post-transfection cells were lysed and equivalent amounts of total protein were analyzed by western blot using a specific antiserum for detection of GCN2. (C) Affinity purified mouse or human GCN2 was incubated for 3 h at 30°C in the absence or the presence of HIV-1 or HIV-2 proteases. Incubation was stopped by addition of SDS-PAGE sample buffer and proteins were analyzed by western blot using a specific antiserum for detection of GCN2. (D) HeLa cell-free extracts were incubated with recombinant HIV-1^Pro^ in the absence or the presence of 2.5 µM SQ for the indicated times. Proteins were analyzed by western blot using specific antisera for detection of GCN2, eIF4GI, PABP or actin as indicated. (E) Purified mouse and human GCN2 GST-fusion proteins were incubated in the presence of recombinant HIV-1^Pro^ for 3 h at 30°C. Proteins were resolved in SDS-PAGE and stained with Coomassie blue. Shown in the lower box is the GCN2 sequence obtained by Edman degradation of the indicated C-terminal fragments. Results are representative of at least three independent experiments.

Next, we tested the ability of HIV-1^Pro^ to cleave GCN2 by using different approaches. First, we checked the cleavage of GCN2 in cells where the protease was expressed in a context in which other HIV-1 proteins were absent. With this aim, we expressed HIV-1^Pro^ in BHK-21 cells by electroporation of an in vitro transcribed RNA bearing the protease-coding region in the context of SV genomic mRNA [25]. As controls we used similar RNAs in order to express the 2A protease of poliovirus (PV-2A^Pro^) or just SV capsid C protein (Figure 2A). At 4 hours post-electroporation, we could observe a marked decrease in the amount of endogenous GCN2 and the appearance of a proteolytic fragment of an apparent molecular weight of 130–140 kDa, accounting for a specific cleavage of GCN2 by HIV-1^Pro^. In contrast, PV-2A^Pro^ was unable to cut GCN2, but, as expected, eIF4GI was proteolytically degraded by both PV-2A^Pro^ and HIV-1^Pro^. We also assayed the proteolytic activity of HIV-1^Pro^ on GCN2 in COS-7 cells transfected with pTM1-derived plasmids and infected with vaccinia virus encoding T7 polymerase (Figure 2B). Co-expression of HIV-1^Pro^ with mouse or human GCN2 resulted in the production of a cleavage fragment similar to the one obtained in BHK-21 cells expressing the protease.

To further study GCN2 proteolytic degradation we performed experiments in which purified HIV-1^Pro^ was added to HeLa cell-free extracts. Complete proteolysis of GCN2 was achieved after only 15 min of incubation, whereas in the same extracts a significant amount of eIF4GI remained intact after 30 min (Figure 2D). The cleavage of both proteins was prevented when the protease was used in the presence of saquinavir. PABP, another known HIV-1^Pro^ substrate, seemed to be processed to a much lower extent and the amount of other proteins such as actin did not experience significant changes.

Moreover, affinity-purifed mouse and human GCN2 were cleaved in vitro after incubation with purified HIV-1^Pro^ and HIV-2^Pro^ (Figure 2C). Both viral proteases rendered GCN2 proteolytic fragments of the same size, suggesting that these proteases directly cleaved both mouse and human GCN2 at the same, or a very close, site.

### Identification of the HIV-1 protease cleavage site on GCN2

Given that HIV-1^Pro^ produced a direct proteolytic break in GCN2, we next tried to identify the precise site/s of cleavage. Based on the size of the major GCN2 fragment obtained by the action of the protease, we constructed bacterial expression plasmids in order to express and purify GST fusion proteins bearing a portion of mouse (aa 419–1024) or human (aa 525–1024) GCN2, which was supposed to contain the cleavage site. We used truncated recombinant proteins due to the low yield of full-length GCN2 protein in bacteria. Protease treatment produced a major carboxy-terminal fragment with an apparent molecular weight of about 67 kDa in both mouse and human proteins (Figure 2E). In the case of the human protein there was a minor additional product of about 73 kDa which could account for a secondary cleavage site. The major 67 kDa polypeptide was subjected to amino-terminal sequencing by using the Edman degradation method, which rendered the amino acid sequences VETVIP for the human protein and IETVIP for the mouse protein, which correspond to a cleavage site between residues 560 and 561 or 559 and 560, respectively. Thus the cleavage sites identified are compatible with the size of the carboxi-terminal GCN2 fragments detected, in the in vivo and the in vitro experiments, using an anti-GCN2 antibody directed against the central portion of the kinase (aa 419–1024).

### HIV-1 protease cleavage abrogates GCN2 activity

**Figure 3:**
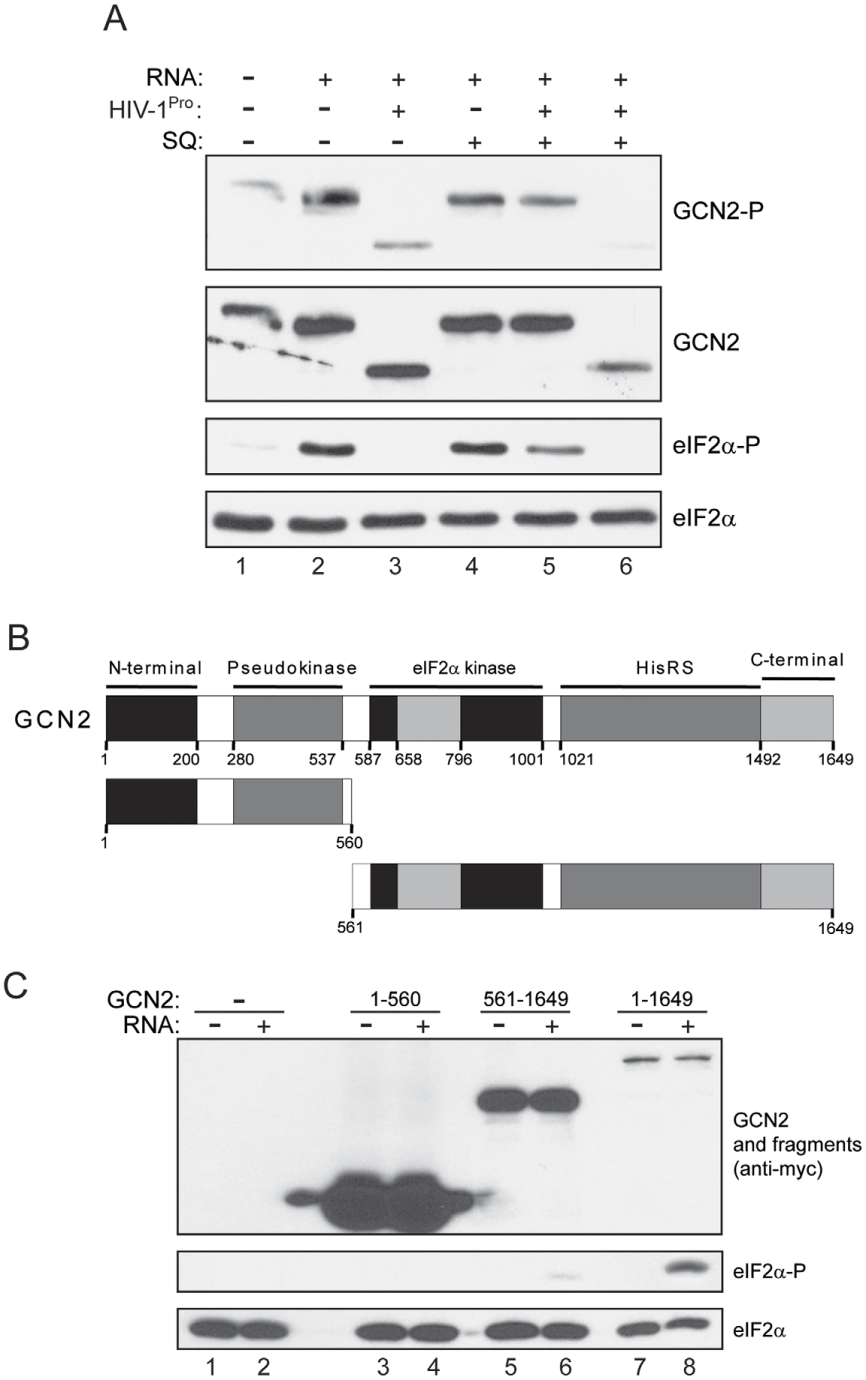
HIV-1 cleavage inhibits GCN2 eIF2alpha kinase activity. (A) Affinity purified human GCN2 was incubated for 30 min at 30°C in the absence or the presence of HIV-1^Pro^ and in the absence or the presence of 2.5 µM SQ, as indicated, prior to being assayed in an eIF2alpha kinase assay (lanes 1–5). In lane 6, SQ was added after incubation of GCN2 with the protease, just before initiating the eIF2alpha kinase assay. HIV-1 RNA was only present during the kinase assay. Incubation was stopped by addition of SDS-PAGE sample buffer and proteins were analyzed by western blot using specific antisera to detect eIF2alpha phosphorylated on serine 51, total eIF2alpha, and phosphorylated and total GCN2. Similar results were obtained from a duplicate experiment. (B) Schematic representation of the structural domains of human GCN2 and the generated GCN2 fragments according to the HIV-1^Pro^ cleavage site. The 1649 amino acid GCN2 sequence is illustrated by a larger box and the figure is drawn to scale. Highlighted domains include the N-terminal (black); the ‘Pseudokinase’ (dark grey) that is related to sub-domains I–XI of eukaryotic protein kinases; the conserved two lobes of the eIF2alpha kinase domain (black), separated by a large insert (light grey); the HisRS-like domain (dark grey); and a C-terminal domain (light grey). The numbers refer to the amino acid residues. (C) In vitro eIF2alpha kinase assay of purified full-length human GCN2 (1–1649) and the generated GCN2 fragments according to the HIV-1^Pro^ cleavage site (1–560 and 561–1649) in the absence or the presence of HIV-1 RNA as indicated. Proteins were analyzed by western blot using specific antisera to detect eIF2alpha phosphorylated on serine 51, total eIF2alpha, and full-length GCN2 or fragments (anti-myc). Similar results were obtained from a duplicate experiment.

To test whether the proteolytic cleavage by HIV-1^Pro^ led to the inactivation of GCN2, we assayed GCN2 in vitro activity after incubation with HIV-1^Pro^. Incubation of GCN2 with HIV-1^Pro^ previous to the eIF2alpha kinase assay rendered a cleaved form of GCN2 which was just slightly phosphorylated and unable to phosphorylate eIF2alpha (Figure 3A, lane 3). To make sure that this effect was due to the proteolytic activity of HIV-1^Pro^, we also performed the assay in the presence of saquinavir, which was added just before the incubation of the protease with GCN2 (Figure 3A, lane 5) or, alternatively, after the incubation of the kinase and the protease, just before initiating the kinase assay (Figure 3A, lane 6). The presence of saquinavir prevented GCN2 cleavage by the viral protease and allowed phosphorylation of the kinase and of eIF2alpha. Thus, we unequivocally concluded that HIV-1^Pro^ activity on GCN2 causes the inactivation of the kinase.

To further investigate the observed effect of HIV-1^Pro^ action on GCN2 activity, we expressed, purified and assayed two polypeptides equivalent to those obtained by HIV-1^Pro^ action on human GCN2, i.e., GCN2 aa 1–560 and aa 561–1649, respectively (Figure 3B). The amino-terminal polypeptide was quite inactive (Figure 3C, lanes 3 and 4), but the carboxi-terminal protein fragment retained some of the ability to phosphorylate eIF2alpha in the presence of HIV-1 RNA (Figure 3C, lanes 5 and 6), although it was significantly lower than the activity shown by the full length GCN2 protein (Figure 3C, lanes 7 and 8). This result corroborates the fact that GCN2 proteolytic cleavage by HIV-1^Pro^ leads to the inactivation of the kinase.

### GCN2 activity inhibits HIV-1 RNA translation

**Figure 4:**
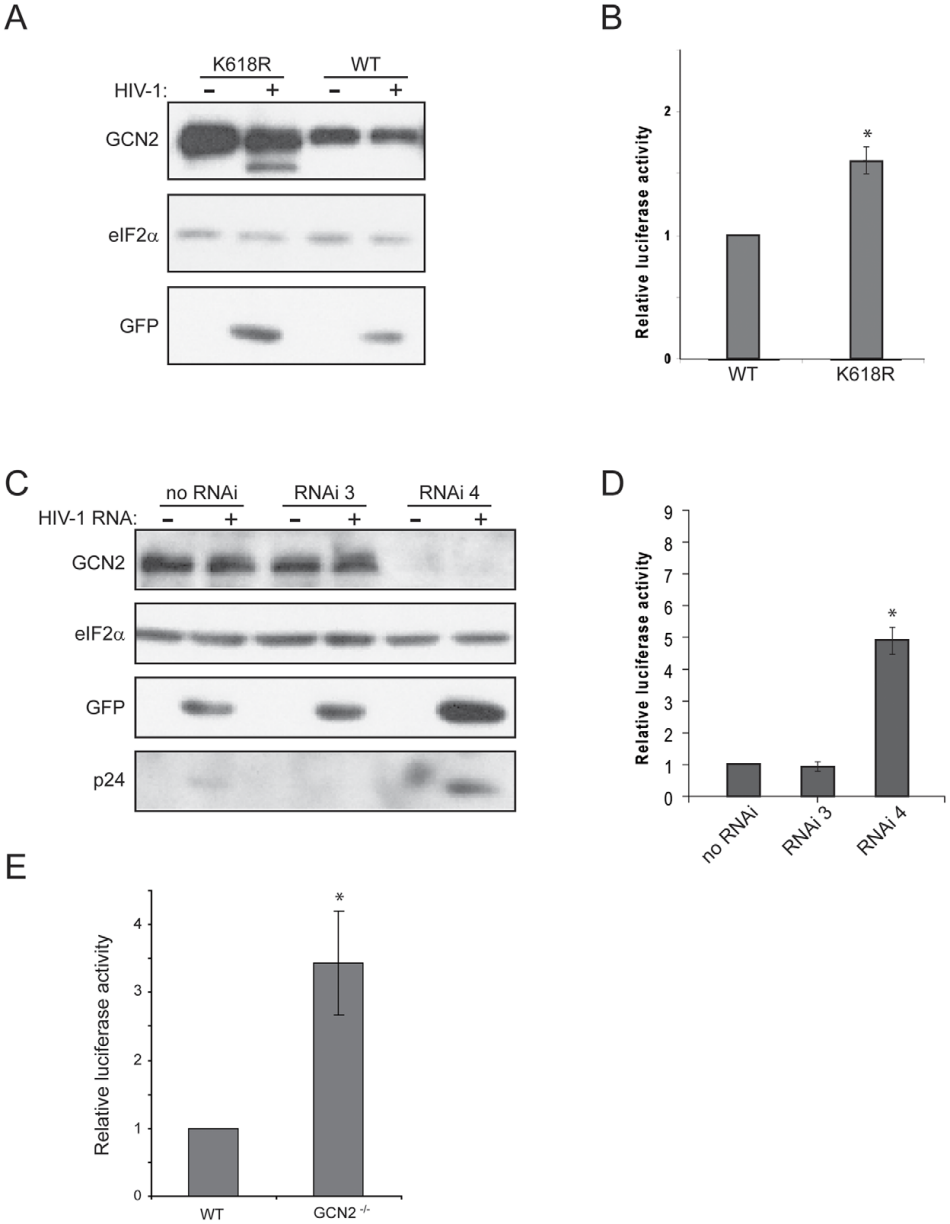
Absence of GCN2 favor HIV-1 protein expression. (A, B) HeLa cells were co-transfected with plasmids encoding wild type GCN2 or the inactive mutant GCN2-K618R and also with a mixture of HIV-1 encoding plasmids pNL4-3.Luc.R-E- and pNL4-3.GFP.R-E-. Cells were harvested 24 h after transfection. Aliquots of cell lysates containing equal amounts of proteins were analyzed by western blot using specific antisera to detect GCN2, eIF2alpha and GFP (A). Luciferase activity was measured, and results are shown as the ratio between the activity in cells over-expressing GCN2-K618R mutant and in cells over-expressing wild type GCN2. * *P*<0.05 vs. WT (B). (C, D) Normal HeLa cells (no siRNA) and HeLa cells stably expressing a control siRNA (siRNA 3) or a specific siRNA for GCN2 knock down (siRNA 4) were transiently transfected with a mixture of HIV-1 encoding plasmids, pNL4-3.Luc.R-E- and pNL4-3.GFP.R-E-. Cells were harvested 24 h after transfection. Aliquots of cell lysates containing equal amounts of proteins were analyzed by western blot using specific antisera to detect GCN2, eIF2alpha, GFP and HIV-1-p24 (C). Luciferase activity was also measured, and results are shown as the ratio between the activity in cells expressing siRNA 3 or siRNA 4 and in normal cells (no siRNA). * *P*<0.01 vs. no RNAi and RNAi 3 (D). (E) Wild type (WT) and GCN2 knock out (GCN2^−/−^) MEF were transiently transfected by electroporation with a pcDNA-based plasmid encoding luciferase protein (pcDNA-Luc), as a control, or with an HIV-1 encoding plasmid, pNL4-3.Luc.R-E-. Cells were harvested 24 h after transfection and luciferase activity was measured in cell extracts. Luciferase activity produced in cells from the HIV-1-encoding plasmid was normalized dividing it by the activity produced in cells from pcDNA-Luc plasmid. Results represent the ratio of normalized luciferase activity between wild type MEFs (set as one) and GCN2^−/−^. * *P*<0.005 vs. WT. Results represent the mean of three to four independent experiments and error bars indicate the standard error (SE).

We wanted to test whether the activity of GCN2 had an effect on the synthesis of HIV-1 proteins. To this end, we used two plasmids encoding the HIV-1 genome in which *luciferase* or *GFP* genes were inserted into the pNL4-3 *nef* gene (pNL4-3.Luc.R-E- and pNL4-3.GFP.R-E-). First, we co-transfected HeLa cells with HIV-1 constructs (pNL4-3.Luc.R-E- or pNL4-3.GFP.R-E-) or pcDNA-Luc, along with the plasmids encoding either wild type GCN2 or the inactive mutant GCN2-K618R. Luciferase activity and GFP expression were significantly higher in cells over-expressing GCN2-K618R when compared with the ones bearing wild type GCN2 (Figure 4 A and B), suggesting a possible negative effect of GCN2 activity on HIV-1 protein synthesis. We also wanted to analyze whether the absence of GCN2 in cells could affect HIV-1 protein expression. To do so, we transfected the plasmids pNL4-3.Luc.R-E- or pcDNA-Luc in MEFs devoid of GCN2 (Figure 4E) or in HeLa cells showing low levels of GCN2 due to the stable expression of a specific interferring RNA (RNAi 4) (Figure 4 C and D). In both cases the relative luciferase expression from the HIV-1 clone was significantly increased (more than 2-fold) in cells with low or absent GCN2 activity when compared with wild type MEFs or HeLa cells bearing a unspecific RNAi. Moreover, in HeLa cells where GCN2 was knocked down we found high levels of GFP and HIV-1 p24 proteins compared to cells bearing normal levels of GCN2 (Figure 4C). These results suggest that GCN2 activity inhibits HIV-1 protein synthesis.

### HIV-1 RNA activates GCN2 in cells

**Figure 5:**
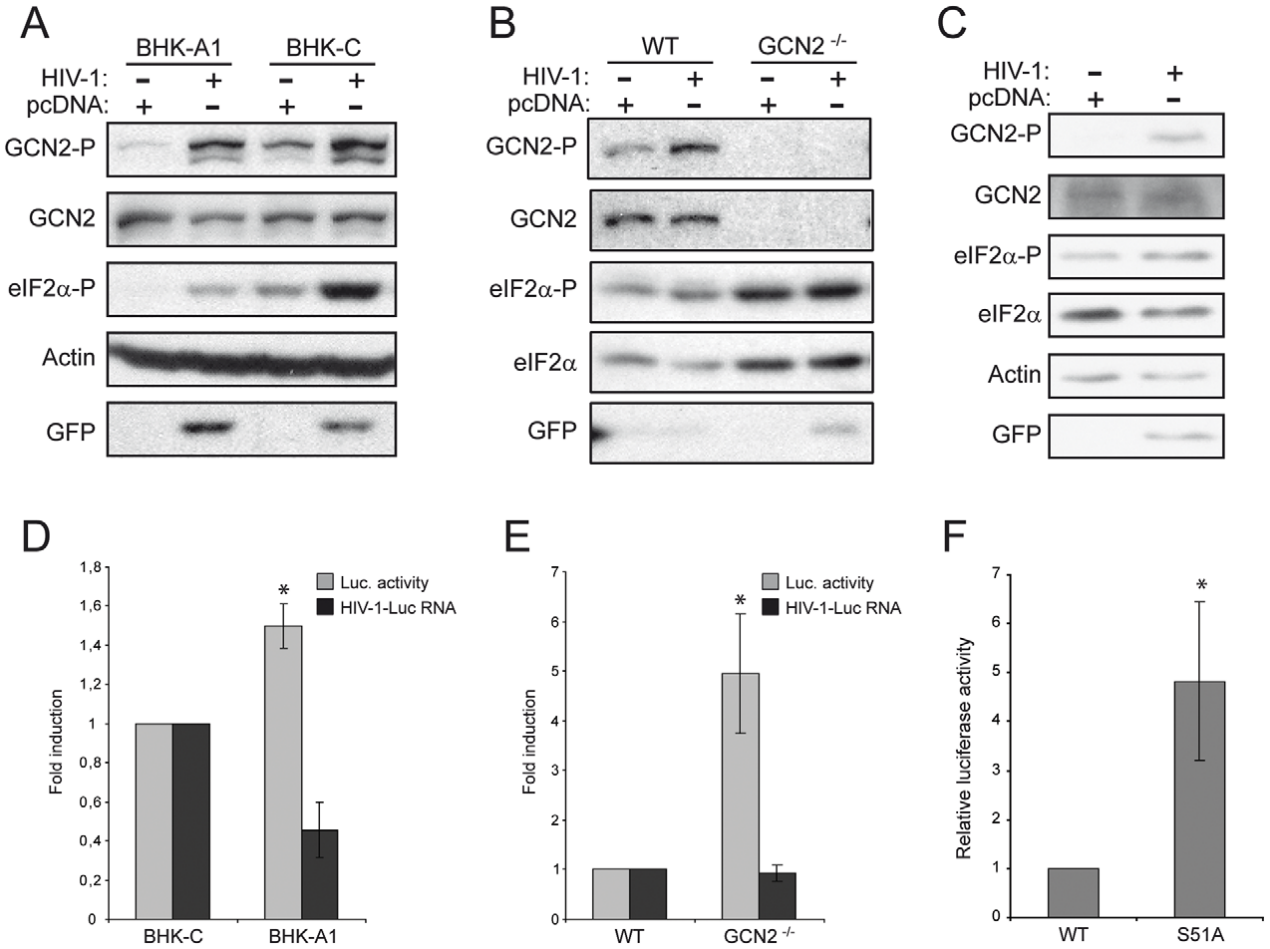
GCN2 is activated in cells expressing HIV-1 RNA. (A) BHK-C and BHK-A1 cells were transfected with a pcDNA-based plasmid encoding luciferase protein (pcDNA-Luc) or with a mixture of HIV-1 encoding plasmids, pNL4-3.Luc.R-E- and pNL4-3.GFP.R-E-. Aliquots of cell lysates containing equal amounts of proteins were analyzed by western blot using specific antisera to detect eIF2alpha phosphorylated on serine 51, phosphorylated or total GCN2, GFP and actin. (B) The same as in (A), but using wild type (WT) and GCN2 knock out (GCN2^-/-^) MEFs. The membrane was probed with different antisera to detect eIF2alpha phosphorylated on serine 51, total eIF2alpha, phosphorylated or total GCN2 and GFP. (C) The same as in (A) and (B), but using HeLa cells. In this case the transfection was done by electroporation. The membrane was probed with different antisera to detect eIF2alpha phosphorylated on serine 51, total eIF2alpha, phosphorylated or total GCN2, GFP and actin. Similar results were obtained from duplicate experiments. (D and E) In samples equivalent to those of the experiments described in (A) and (B) where cells were transfected with pNL4-3.Luc.R-E- plasmid, luciferase activity was measured and HIV-1 RNA quantified. Relative values of both are shown as the ratio between GCN2^-/-^ MEFs or BHK-A1 and the corresponding control cells (wild type MEFs or BHK-C), whose value was set as one. Data are expressed as mean ± SE. * *P* < 0.05 vs. BHK-C (D) or WT (E). (F) Wild type (WT) and S51A MEFs were transiently transfected by electroporation with a pcDNA-based plasmid encoding luciferase protein (pcDNA-Luc), as a control, or with an HIV-1 encoding plasmid, pNL4-3.Luc.R-E-. Cells were harvested 24 hr after electroporation and luciferase activity was measured in cell extracts. Luciferase activity produced in cells from the HIV-1-encoding plasmid was normalized dividing it by the activity produced in cells from pcDNA-Luc plasmid. Results represent the ratio of normalized luciferase activity between wild type MEFs (set as one) and S51A MEFs. * *P* < 0.01 vs. WT.

We performed transfection with the above mentioned HIV-1 RNA-encoding plasmids in a BHK-21 cell line that over-expresses the regulatory subunit of PP1 phosphatase, GADD34, rendering cells with low levels of phosphorylated eIF2alpha, BHK-A1 (Figure S1). In these cells the expression of HIV-1 constructs induced a significant phosphorylation of GCN2 and the subsequent increase in the levels of phosphorylated eIF2alpha, which was also observed in MEFs and HeLa cells (Figure 5 A, B and C) transfected with plasmids encoding HIV-1 RNA. It is also noteworthy that the amount of GFP, as a reporter of viral protein synthesis, was higher in the BHK-A1 cell line and in GCN2 knock out (GCN2−/−) than in wild-type MEFs (Figure 5 A and B). We also observed that luciferase activity expressed from a HIV-1 RNA was higher in BHK-A1 cells, in cells expressing a non-phosphorylatable form of eIF2alpha and in GCN2−/− MEFs than in the respective control cells, BHK-C and wild type MEFs (Figure 5 D, E and F). This increase should be caused by differences in translation, given that the levels of the luciferase-encoding HIV-1 RNA did not rise in those cells (Figure 5 D and E). Thus we can conclude that HIV-1 RNA activates GCN2, which in turn phosphorylates eIF2alpha leading to the inhibition of HIV-1 RNA translation.

## Discussion

In eukaryotic cells the translation of mRNAs into proteins is mainly regulated at the initiation step, where regulation of the eIF2 function by phosphorylation of its alpha subunit by specific protein kinases (eIF2alpha kinases) plays a critical role, given that eIF2alpha phosphorylation leads to a general inhibition of protein synthesis. Synthesis of viral proteins relies on the host cell translation machinery and thus is similarly regulated. In fact, phosphorylation of eIF2alpha by activated eIF2alpha kinases PKR and GCN2 has been shown to be an important mechanism for inhibition of viral protein synthesis and viral cycle progression [14], [26].

Here we report that the presence of HIV-1 RNA increases GCN2 eIF2alpha kinase activity in vitro and that this increase is dependent on the m2 motif in the HisRS-related domain of the kinase. This effect of a viral RNA on GCN2 activity was previously observed for the genomic RNA of Sindbis virus [14], suggesting that GCN2 is an RNA binding protein which senses the presence in the cells of viral RNAs through its HisRS-related domain in order to activate an antiviral response. Unlike the previous report on Sindbis virus [14], we have not yet identified the RNA sequences of HIV-1 RNA responsible for the activation of GCN2. As shown in this work, the introduction of HIV-1 RNA by transfection of cells with an HIV-1-encoding cDNA produced the activation of GCN2 and a subsequent increase in the levels of phosphorylated eIF2alpha, similar to what was observed in vitro. The consequence of this fact is a reduction in the levels of proteins whose synthesis depends on the translation of HIV-1 RNA and, finally, it could lead to diminished virus production. Its is not surprising that phosphorylation of eIF2alpha led to the inhibition of the viral protein synthesis, as has been widely proven that over-expression and activation of the eIF2alpha kinase PKR produces the same effect [17], [27]. The novelty of our results is that the inhibitory phenomenon is due to the activation of GCN2, given that it has been observed by comparison of HIV-1 protein expression between wild type and GCN2 knock out cells, and in BHK-21 cells (control and over-expressing an eIF2alpha phosphatase activator) in which PKR expression is extremely low compared with other cell types [28]. Thus, the absence of GCN2 in normal cells or the maintenance of low levels of phosphorylated eIF2alpha in cells with a very low presence of PKR favors the expression of HIV-1 proteins.

The relevance of PKR activation and eIF2alpha phosphorylation in HIV-1 protein expression and viral cycle progression is illustrated by a number of countermeasures employed by the virus to prevent PKR response, including viral products and cellular proteins [17]. Therefore, PKR is inhibited by the presence of large amounts of viral TAR RNA and by binding of the HIV-1 Tat protein [29], but also by direct interaction with two cellular RNA binding proteins, the TAR RNA binding protein (TRBP) [30] and adenosine deaminase acting on RNA 1 (ADAR1) [27], [31]. These mechanisms seem to make the innate antiviral response ineffective and allow the virus to replicate in permissive cells. Here we show that in cells (MT-2 and PBMC) infected with HIV-1, GCN2 is proteolytically degraded and that its proteolytic cleavage is prevented by the presence of the specific inhibitor of HIV-1^Pro^ saquinavir. Moreover, the presence of saquinavir increased eIF2alpha phosphorylation, probably due to the activation of uncleaved GCN2. Since GCN2 levels seem to increase with PHA treatment and HIV-1 infection, we can speculate that activation of cells and HIV-1 infection can promote the establishment of an antiviral state that includes GCN2 activation and eIF2alpha phosphorylation, being in part counteracted by the proteolytic degradation of GCN2. Theses findings are consistent with previous reports showing that HIV-1^Pro^ cleaves other proteins involved in the regulation of protein synthesis, such as the translation initiation factors eIF4GI and PABP [20], [24]; this cleavage inhibits cellular protein synthesis, but allows translation of viral RNA [32] which in turn also needs active eIF2alpha. In good agreement with these observations, is the fact that during HIV-1 infection the breakage of GCN2 showed kinetics coinciding with those observed for eIF4GI.

Our results demonstrate that HIV-1^Pro^ cleavage of GCN2 renders an inactive eIF2alpha kinase, although, according to the break site identified, the C-terminal portion of the protein retains the eIF2alpha kinase and HisRs-related domains, responsible for the activity and the activation of the protein, respectively. In fact, it seems that this fragment has some residual activity compared to the full-length protein. This apparent discrepancy could be explained by the possible existence of an additional minor cleavage site in the kinase or the HisRS-related domain. Due to the low sequence identity between HIV-1^Pro^ known substrates and also to the lack of an obvious consensus cleavage motif [33], [34], it is difficult to say if GCN2 is a good substrate for the viral protease, although the cleavage site in GCN2 meets the apparent requirement of hydrophobic amino acids. Our results in HeLa cell-free extracts showed that GCN2 was completely broken after 15 min of protease treatment, whereas after 30 min significant amounts of eIF4GI and, especially, PABP remained intact, suggesting that GCN2 is an excellent substrate for the protease and that its specific breakage could favor HIV-1 cell cycle progression.

All these findings taken together would point to the following plausible scenario for the role of GCN2 in the antiviral response against HIV-1: in the late phase of the viral life cycle, when there is a huge production of viral mRNA to be translated into proteins for virion assembly and maturation, viral RNAs could activate GCN2, which subsequently would phosphorylate eIF2alpha, leading to the inhibition of viral protein synthesis. The translation of some viral mRNAs brings about the appearance of HIV-1^Pro^, which inactivates GCN2 by proteolytic cleavage, preventing phosphorylation of eIF2alpha and allowing the translation of viral proteins and the progression of the viral cycle. It is possible that the discovery of specific activators of GCN2 could help prevent virus replication and the spread of infection.

## Materials and Methods

### Plasmids and in vitro transcription

The pTM1-HIV-1 PR plasmid containing a cDNA fragment encoding the gene of HIV-1 protease in the pTM1 vector [35] has been described previously [20]. The plasmids pT7SV-HIV-1PR, pT7SV-2Apro and pT7SVwt were also described previously [25]. The cDNA containing the entire open reading frame of human GCN2 was subcloned into the plasmid pcDNA3.1/Myc-His (Invitrogen) by PCR amplification combined with restriction endonuclease cuts from different cDNA partial clones. This cDNA was used to generate the plasmids encoding the fragments of human GCN2 corresponding to amino acids 1–560 and 561–1649. The plasmids pGST-mGCN2 (419–1024) and pGST-hGCN2 (525–1024) were constructed by cloning the PCR-amplified cDNA fragments encoding the amino acids 419 to 1024 of mouse GCN2 and 525 to 1024 of human GCN2, respectively, into the plasmid pGEX-4T (GE Healthcare). The HIV-1 cDNA cloned into the plasmid pBH10 [36] was subcloned into the plasmid BlueScript KS (+/−) (Stratagene) in order to allow its transcription from a T7 promoter (pBS-HIV-1).

Plasmids pT7SV-HIV-1PR, pT7SV-2Apro, pT7SVwt and pBS-HIV-1 were linearized and in vitro transcribed using the T7 RNA polymerase kit (Promega) in order to produce capped recombinant genomic Sindbis virus (SV) mRNAs and genomic-length HIV-1 RNA.

The following reagent was obtained through the AIDS Research and Reference Reagent Program, Division of AIDS, NIAID, NIH: pNL4-3.Luc.R-E- from Dr. Nathaniel Landau [37], [38]. An equivalent construct in which the firefly *luciferase* gene was replaced by the green fluorescent protein (GFP) gene (pNL4-3.GFP.R-E-), was kindly supplied by Dr. Gustavo del Real (Centro Nacional de Biotecnología, Madrid, Spain).

### Cell culture, virus infection and transfection

MEF wild type, GCN2 knock out [39] or with a homozygous knock-in mutation for eIF2alpha (S51A) [40], HeLa, COS-7, BHK-21 and HEK 293T cell lines were grown in Dulbecco's modified Eagle's medium (DMEM) containing 10% (v/v) foetal bovine serum (FBS), 2 mM glutamine, antibiotics and non-essential amino acids. MT-2 cells (human T-cell leukemia virus type 1 (HTLV-1)-infected cell line) were maintained in complete RPMI 1640 growth medium (Biochrom AG) supplemented with 5% FBS, 2 mM glutamine, 1% ampicillin, 1% cloxacillin, 0.32% gentamicin. Human Peripheral Blood Mononuclear Cells (PBMC) were derived from healthy voluntary donors, and obtained from leukophoresed blood by Ficoll^TM^ gradient (Pharmacia Fine Chemicals, Uppsala, Sweden) and elutriated by centrifugation. After washing with phosphate buffered saline (PBS), cells were seeded in RPMI 1640 medium (Biochrom AG) with 10% FBS, 1% L-glutamine, antibiotics, and 50 IU/ml IL-2. PBMC were stimulated for 48 h with phytohemaglutinine (2 µg/ml) and interleukin 2 (IL-2) (100 IU/ml). All cell lines were cultured at 37°C in a 5% CO_2_ atmosphere. PBMC were kindly provided by the Spanish HIV BioBank integrated in the Spanish AIDS Research Network (RIS) 1 [41].

PBMC and MT-2 cells were infected or mock-infected with 10 or 100 ng of p24^gag^ per 10^6^ cells of HIV-1_NL4–3_ for 2 h (100 ng of p24^gag^ per 10^6^ cells has been evaluated to be equivalent to 1–2 viral particles per cell) [42], washed three times and incubated in fresh medium. At 1 to 5 days post-infection (dpi), cells were harvested and washed 3 times with PBS, and the dry pellet was stored at −80°C before lysis.

BHK-21 cells were electroporated with 30 µg of recombinant SV genomic RNA in a final volume of 50 µl as described in [25]. Electroporated cells were maintained in growth medium, and, at 4 h post-electroporation, metabolically labelled with [^35^S]Met/Cys mixture (Promix, Amersham), as described previously [14] and lysed in sample buffer. Coupled infection/DNA transfection of COS-7 cells with recombinant vaccinia virus (vvT7), pTM1-derived plasmids and plasmids encoding human and mouse GCN2, have previously been described in detail [43].

For transient transfections, HEK 293T and HeLa cells were plated on 60-mm dishes and transfected with plasmids (6–12 µg/dish) using Lipofectamine^TM^ and Plus^TM^ Reagents (Invitrogen) according to the manufacturer's instructions. Similarly, BHK-21 and MEF cells were transfected using the cationic polymer reagent jetPEI^TM^ (PolyPlus-transfection) according to the manufacturer's instructions.

### Virus production and titration

Virus stocks were prepared by amplification of HIV-1_NL4-3_ in MT-2 cell line (ATCC) or PHA+IL-2 activated PBMC. Physical titers of viral stocks were evaluated by quantification of HIV p24^gag^ by ELISA kit (Innotest HIV-1 antigen mAb; Innogenetics) [44].

### Lentivirus and stable transduction of shRNAs

The following targeted shRNAs in lentiviral construct plasmid pLKO.1-Puro [45] were purchased from Sigma-Aldrich: GCN2 (XM_496066) Clone 1 TRCN0000078648, Clone 2 TRCN0000078649, Clone 3 TRCN0000078650, Clone 4 TRCN0000078651, Clone 5 TRCN0000078652. Lentiviruses were produced by co-transfection of HEK293T Phoenix cells with pLKO.1-Puro shRNA-encoding plasmids, pMD2G (Addgene) and pSPAX2 (Addgene), using jetPEI^TM^ (PolyPlus-transfection). Viruses were harvested at 24 and 48 h post-transfection and infections of HeLa cells were carried out in the presence of 8 μg/ml of polybrene (Sigma-Aldrich). Following transduction, cells were selected with 1 μg/ml puromycin (Sigma-Aldrich).

### Protein purification

For purification of GST fusion proteins, *Escherichia coli* BL21 cells were transformed with plasmids pGST-mGCN2 (419–1024) and pGST-hGCN2 (525–1024), lysed by sonication, and the fusion proteins were then purified as previously described [46] using glutathione-sepharose (GE Healthcare). Purification of Myc- and 6xHis-tagged GCN2 full-length, GCN2-K618R, GCN2-m2 and GCN2 fragments was done as previously described [14]. HIV-1^Pro^ was provided by I. Pichova through the Centralised Facility for AIDS Reagents. Purified HIV-2 protease (HIV-2^Pro^) [47] was obtained through the NIH (National Institutes of Health) AIDS Research and Reference Reagent Program, Division of AIDS, NIAID (National Institute of Allergy and Infectious Diseases), NIH, Bethesda, MD, U.S.A., from Dr Bret Shirley and Mr Michael Cappola (Boehringer Ingelheim Pharmaceuticals).

### Protease cleavage assays

Crude HeLa S10 extracts were incubated with 2.5 ng/µl of recombinant HIV-1^Pro^ and reactions were stopped by adding sample buffer. In other experiments, purified preparations of mouse or human Myc- and 6xHis-tagged GCN2 were incubated with 2.5 ng/µl of recombinant HIV-1^Pro^, or HIV-2^Pro^ in cleavage buffer (50 mM sodium phosphate, pH 6.0, 25 mM NaCl, 5 mM EDTA and 1 mM DTT) for 3 h at 30°C. In order to map the cleavage sites of HIV-1^Pro^ on GCN2, 50 µg of recombinant GST–tagged mouse or human GCN2 protein were incubated with 1 µg of recombinant HIV-1^Pro^ in a total volume of 50 µl for 3 h at 30°C in cleavage buffer. Cleavage products were separated by SDS-PAGE, transferred to a PVDF membrane (Immobilon®–P, Millipore) and then subjected to automated Edman degradation with an Applied Biosystems Procise Sequencer in the Proteomics Service of Centro de Investigaciones Biológicas (CSIC).

### *In vitro* kinase activity

Affinity purified GCN2 wild type and mutants (m2 and K618R) were assayed for their ability to phosphorylate eIF2alpha in the presence or the absence of HIV-1 RNA, as previously described [14]. In some experiments ^32^P-γ-ATP was included in the kinase reaction mix and phosphorylated eIF2alpha was detected by autoradiography of the corresponding SDS-PAGE dried gel. In other kinase assays only non-radiactive ATP was used and phosphorylated eIF2alpha and GCN2 were detected by western blot using phosphospecific antibodies.

### Western blotting

Proteins subjected to SDS-PAGE were transferred to a PVDF membrane (Immobilon®–P, Millipore) for western blot analysis. Western blots were performed using the following antibodies: mouse anti-myc (Sigma); rabbit anti-eIF2alpha phospho-Ser51 (Cell Signaling); rabbit anti-eIF2alpha (Santa Cruz); rabbit anti-GCN2 phospho-Thr898 (Cell Signaling, Abcam); rabbit anti-GCN2 [8]; eIF4GI anti-serum raised against peptides derived from the C-terminal region of the human eIF4GI [43]; rabbit anti-sera raised against the C- and N-terminal region of human PABP [48]; and mouse ascites of a monoclonal antibody against HIV-1 p24 antigen (Centralised Facility for AIDS Reagents, NIBSC, Potters Bar, Herts., U.K.). Goat anti-(rabbit IgG) and goat anti-(mouse IgG) antibodies coupled to peroxidase (Promega) were also used. Immunoreactive bands were visualized by using ECL^TM^ and ECL Plus Western Blotting Detention Systems (GE Healthcare) and autoradiography (Agfa Curix RP2 Plus).

### Luciferase activity determination

After different times post-transfection, cells were washed twice with PBS and lysed using Passive Lysis Buffer (Promega). After removal of cell debris, equal amounts of total protein were used to measure luciferase activity (Luciferase Assay System, Promega) in the lysates.

### RNA extraction, reverse transcription and real-time PCR

Total RNA was extracted using the SV Total RNA Isolation System (Promega), following the manufacturer's instructions. For determination of luciferase mRNA expression, 1 µg of total RNA was reverse-transcribed in a 20 µl reaction mixture by using a Reverse Transcription System (Promega) and random primers following the manufacturer's instructions. The LightCycler® FastStart DNA Master^PLUS^ SYBR Green I system (Roche) was used for real-time PCR amplification/quantification using 2 µl of first strand cDNA reaction as a template. The level of GAPDH mRNA in each sample was determined in order to normalize for differences in total RNA amounts. The data were derived from at least three independent reverse-transcription reactions and real-time PCR performed in duplicate. Data analysis to determine relative luciferase mRNA expression was performed according to the 2^-ΔΔCT^ method [49]. Primers were designed to amplify fragments in the open reading frame of luciferase (nucleotides 1343-1540) and GAPDH (nucleotides 493–775).

### Statistical Analysis

All data are reported as means ± SE of the results obtained from at least three independent experiments. Data were analyzed by two-tailed, unpaired Student's t test. Differences were considered significant when *P*<0.05.

## Supporting Information

Figure S1 **GCN2 and PKR expression and eIF2alpha kinase response in BHK-C and BHK-A1 cells.** (A) BHK-C and BHK-A1 cells were maintained for 1 h in normal growth medium containing or not thapsigargin (Tg) or in growth medium without methionine (-Met). Aliquots of cell lysates containing equal amounts of proteins were resolved into 10% SDS-PAGE and transferred to a PVDF membrane. The membrane was probed with different antisera to detect eIF2alpha phosphorylated on serine 51, phosphorylated or total GCN2 and actin. (B) BHK-C and BHK-A1 cells or wild type (WT), GCN2 knock out (GCN2^-/-^) and PKR knock out (PKR^-/-^) MEFs were maintained in normal growth medium before lysis. Aliquots of cell extracts containing equal amount of proteins were resolved into 10% SDS-PAGE and transferred to a PVDF membrane. The membrane was probed with different antisera to detect GCN2, PKR and actin. Results are representative of at least three independent experiments.
(TIF)
