# Supplementary figures and images for: GCN2 Has Inhibitory Effect on Human Immunodeficiency Virus-1 Protein Synthesis and Is Cleaved upon Viral Infection
Source: PLoS One. 2012 Oct 23;7(10):e47272. doi: 10.1371/journal.pone.0047272 (PMC3479103; doi:10.1371/journal.pone.0047272)

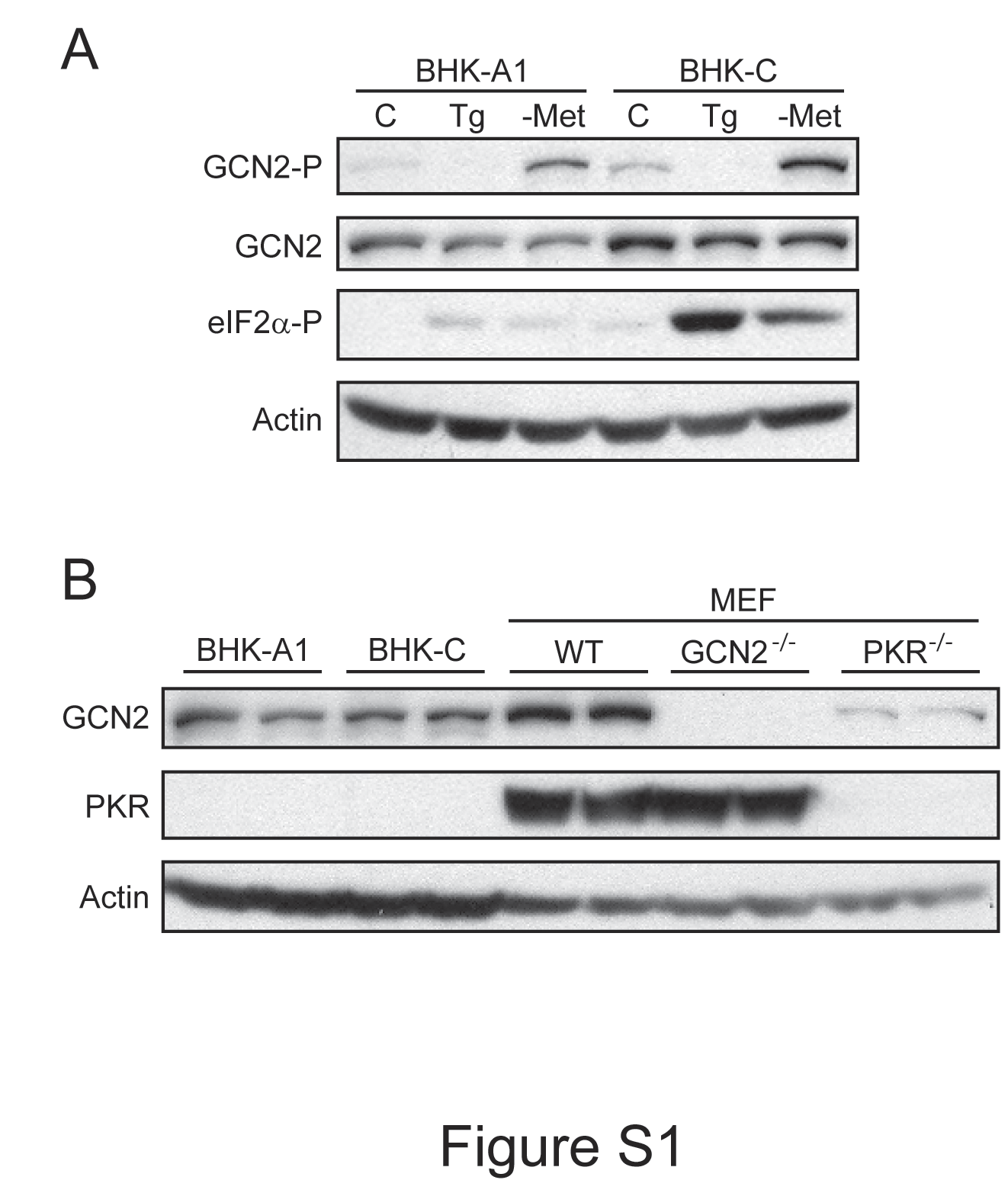

Supplement: Figure S1 — GCN2 and PKR expression and eIF2alpha kinase response in BHK-C and BHK-A1 cells. (A) BHK-C and BHK-A1 cells were maintained for 1 h in normal growth medium containing or not thapsigargin (Tg) or in growth medium without methionine (-Met). Aliquots of cell lysates containing equal amounts of proteins were resolved into 10% SDS-PAGE and transferred to a PVDF membrane. The membrane was probed with different antisera to detect eIF2alpha phosphorylated on serine 51, phosphorylated or total GCN2 and actin. (B) BHK-C and BHK-A1 cells or wild type (WT), GCN2 knock out (GCN2-/-) and PKR knock out (PKR-/-) MEFs were maintained in normal growth medium before lysis. Aliquots of cell extracts containing equal amount of proteins were resolved into 10% SDS-PAGE and transferred to a PVDF membrane. The membrane was probed with different antisera to detect GCN2, PKR and actin. Results are representative of at least three independent experiments. (TIF) [file pone.0047272.s001.tif]
